# Generation of human-induced pluripotent stem cells from a patient with homozygous I1234V mutation of cystic fibrosis

**DOI:** 10.5339/qmj.2024.qitc.4

**Published:** 2024-04-08

**Authors:** Mohamed M. Emara, Merlin Thomas, Mona Al Langawi, Michail Nomikos, Hanaa Mousa, Soha Aboukhalaf, Nadin H Abouzeid, Yasemin AlShanableh, Maryam K Al Thani, Yehia Y. Hussein, Nuha T. Swaidan, Yasmin Elsharabassi

**Affiliations:** 1 Basic Medical Sciences Department, College of Medicine, QU Health, Qatar University, Doha, Qatar Email: memara@qu.edu.qa; 2 Department of Chest, Hamad Medical Corporation, Doha, Qatar; 3 Weill Cornell Medicine, Doha, Qatar

**Keywords:** Cystic fibrosis, homozygous I1234V mutation, Induced pluripotent stem cells

## Introduction

Cystic fibrosis (CF) is an inherited autosomal recessive disorder. The most predominant mutation among Qatari patients with CF is the homozygous I1234V mutation, which is more prevalent in a Bedouin tribe.^1^ There are no reliable models of this mutation available to study the pathogenesis of CF; therefore, discovery of an effective treatment for the disease is ongoing.

## Methods

We conducted this study to generate patient-specific induced pluripotent stem cells (iPSCs) from an adult Qatari CF patient with the above-mentioned mutation to fully characterize these cells and eventually use them in disease modeling. The study was ethically approved by the Institutional Review Board of Hamad Medical Corporation (HMC) and Qatar University (QU). Blood samples (10 ml) were collected at HMC from the patient with CF and a matched healthy control and processed at QU based on Takahashi et al.^2^

## Results

**Figure 1.**
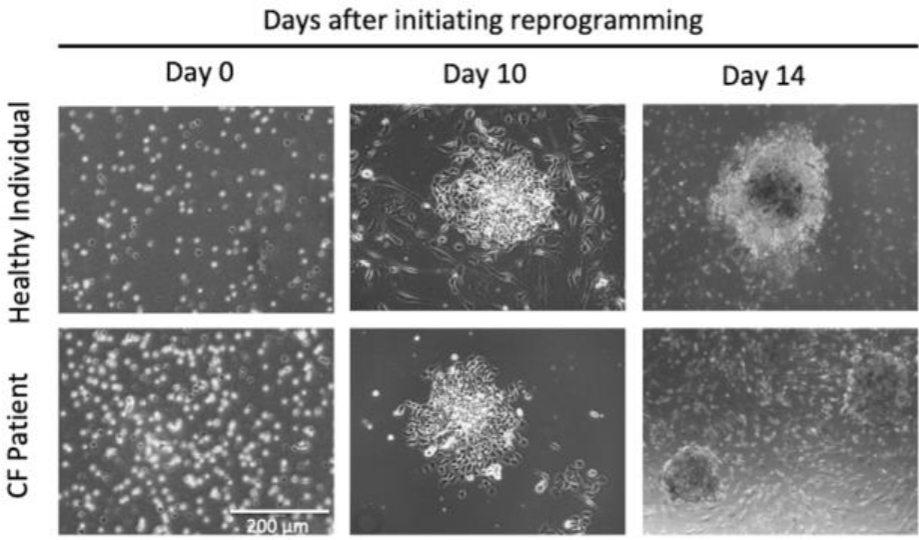
Morphological changes and aggregation of PBMCs indicating iPSC generation. Microscopic images showing the first stages of emerging iPSC colonies.

**Figure 2.**
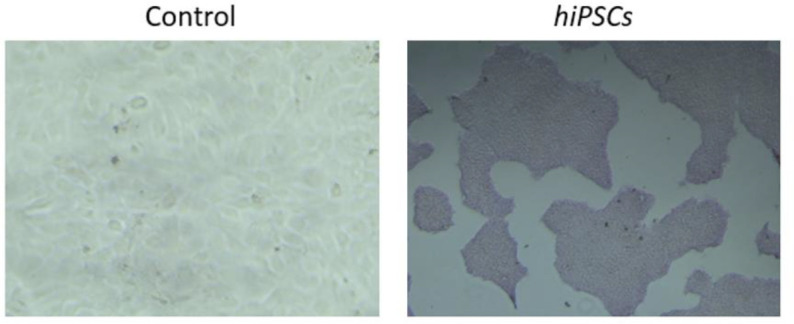
The images show the difference in alkaline phosphatase activity when staining fibroblast cells as a control on the left and the generated iPSC colonies on the right.

The emerging iPSC colonies were monitored daily until we observed the typical morphology of undifferentiated human embryonic stem cell-like colonies and/or commercial iPSCs (Figure 1). These colonies were fully characterized by different molecular and cell biology techniques and showed similar characteristics of the commercial ones. Indeed, different known pluripotent protein markers (Nanog, Oct4, SOX2, SOX17, and Brachyury) were efficiently expressed as assessed by Western blot analysis. The presence of these pluripotent markers was also confirmed using fluorescence microscopy. In addition, the generated cells markedly expressed alkaline phosphatase, which is a key marker of pluripotent stem cells (Figure 2). Finally, the clones maintained the normal integrity of the 46, XY karyotype chromosome.

## Conclusion

These results indicate that we have succeeded for the first time in generating CF-I1234V-hiPSC colonies that have the typical pluripotent cell morphology and carry all the molecular characteristics of pluripotent stem cells. The generation of this cell model could be used to tailor personalized treatment specifically for Qatari CF patients with this mutation.

## Conflict of Interest

The authors have no conflict of interest to disclose.

## Ethical Approval

The research described in this paper has received ethical approval from Hamad Medical Cooperation: MRC-03-20-050 and Qatar University: QU-IRB 1247-EA/20 Institutional Review Boards (IRB) in accordance with established ethical guidelines and regulations.

## References

[1] Hammoudeh S., Gadelhak W., AbdulWahab A., Al-Langawi M, Janahi IA (2019;). Approaching two decades of cystic fibrosis research in Qatar: A historical perspective and future directions. Multidisciplinary Respiratory Medicine.

[2] Takahashi K, Tanabe K, Ohnuki M, Narita M, Ichisaka T, Tomoda K (2007;). Induction of pluripotent stem cells from adult human fibroblasts by defined factors. Cell.

